# Development of Test Methods to Evaluate the Printability of Concrete Materials for Additive Manufacturing

**DOI:** 10.3390/ma15186486

**Published:** 2022-09-19

**Authors:** Youssef Mortada, Malek Mohammad, Bilal Mansoor, Zachary Grasley, Eyad Masad

**Affiliations:** 1 Mechanical Engineering Program, Texas A&M University at Qatar, Doha P.O. Box 23874, Qatar; 2 Department of Materials Science and Engineering, Texas A&M University, 575 Ross Street, College Station, TX 77843, USA; 3 Oryx Universal College in Partnership with Liverpool John Moores University, Doha P.O. Box 12253, Qatar; 4 Zachry Department of Civil Engineering, Texas A&M University, College Station, TX 77843, USA

**Keywords:** additive manufacturing, printability, ultrasonic pulse velocity, 3D concrete printing

## Abstract

This study proposes test methods for assessing the printability of concrete materials for Additive Manufacturing. The printability of concrete is divided into three main aspects: flowability, setting time, and buildability. These properties are considered to monitor the critical quality of 3DCP and to ensure a successful print. Flowability is evaluated through a rheometer test, where the evolution of shear yield strength is monitored at a constant rate (rpm), similar to the printer setup. Flowability limits were set based on the user-defined maximum thickness of a printed layer and the onset of gaps/cracks during printing. Setting time is evaluated through an ultrasonic wave pulse velocity test (UPV), where the first inflection point of the evolution of the UPV graph corresponds to the setting time of the concrete specimen. The results from this continuous non-destructive test were found to correlate with the results from the discrete destructive ASTM C-191 test for measuring setting time with a maximum difference of 5% between both sets of values. Lastly, buildability was evaluated through the measurement of the early-age compressive strength of concrete, and a correlation with the UPV results obtained a predictive model that can be used in real-time to non-destructively assess the material buildability. This predictive model had a maximum percentage difference of 13% with the measured values. The outcome of this study is a set of tests to evaluate the properties of 3D printable concrete (3DP) material and provide a basis for a framework to benchmark and design materials for additive manufacturing.

## 1. Introduction

Additive manufacturing (AM) has received a significant amount of interest and research in the past few decades, especially with the overwhelming belief that it is the main cornerstone in the leap to Manufacturing 4.0 [1]. This interest has extended into virtually every major industry such as the automotive, aerospace, medicinal, and even the construction industry. In the construction industry, the majority of interest has been directed at concrete printing through material extrusion mechanisms, which have generated the most research in the field [2]. This strong interest in AM is due to its ability to address many of the limitations associated with the current concrete industry by providing greater flexibility in geometrical design, higher speeds of construction, higher sustainability impact, the elimination of formwork usage, and lower waste production during manufacturing. Additionally, AM has the potential to decrease the total cost of construction by 35–60% [3,4].

Printability in AM refers to the need for certain conditions to be met in order to ensure the successful fabrication of the required print. These conditions vary from one material to another, and in concrete AM they focus on material properties that are less important for conventional concrete construction involving casting in formwork. These properties include flowability, buildability, and initial setting time. Flowability is defined as the ability of the material to be pumped and extruded without any breakages and disruptions in the flow while retaining its deposited shape. Buildability, on the other hand, is the ability of the deposited layers to gain enough strength to withstand the weight of subsequent layers and avoid structural collapse [5]. Setting time is defined as the elapsed time from mixing necessary for sufficient hydrate formation to enable a solid capable of bearing a certain magnitude of shear stress. Although setting time is already a property of interest for traditional concrete, its importance in the AM of concrete is higher. Traditional concrete has an initial setting time of approximately 4 h, which is convenient for the traditional method to provide sufficient time to place the concrete in the molds. As concrete is a time-dependent material, its early strength gain is directly proportional to the setting time. Versus conventional concrete, the setting time of concrete for AM applications needs to be reduced greatly in order to allow for the ability of layers to be deposited onto one another without collapse [6]. These three properties (flowability, setting time, and buildability) focus on the crucial stages of printing and would be of main consideration prior to mixing in order to make sure they fit the needs of the desired print. Therefore, they are introduced as the controlling properties that are essential for printing to occur and are the main focus of this study. 

Flowability has mainly been investigated in the literature as it relates to material characterization through the identification of the rheological properties of the material in question [7,8,9,10]. These investigations vary in their approaches, with many of them characterizing the behavior of fresh 3D printable (3DP) concrete with a Bingham model to identify the corresponding rheological properties of the material [7,8,9,10]. However, there have not been any standardized testing methods and/or procedures that were used uniformly throughout these investigations, such that various empirical methods and rheological testing procedures were used. Moreover, these studies have not aimed at assessing whether or not the material would be printable and if the flowability can be monitored and adjusted, if need be, during printing. On the contrary, these investigations mainly focus on the effects of mixture variables (such as water content, chemical additives, and aggregates), as well as external disturbances, such as vibrations, on the rheological properties of the material [9,10,11,12,13]. Therefore, relating rheological parameters to the quality of printing has not been addressed properly, and such a relationship could establish an important connection in assessing printability. 

Monitoring the setting time of 3DP concrete is important since fluctuations in the setting time of deposited concrete can greatly affect the printing process, as well as the integrity of the print. These fluctuations in setting time can stem from the use of chemical admixtures, such as accelerators and retarders, while printing and/or from variable on-site printing conditions such as ambient temperature and humidity [12]. If the setting time is greatly reduced, then there is a possibility that the flow of material from the mixer to the nozzle is not continuous, which would cause breakages and defects in the extruded layers, as well as issues related to subsequent layer bonding. On the other hand, if the setting time is delayed with the addition of admixtures such as retarders, then the extruded flow would not be able to gain enough strength in time to support the deposition of additional layers and thus the integrity of the print is at risk. At present, the ASTM C-191 test using the Vicat needle is the most common method used to determine the setting time of concrete. This is a destructive test that cannot be used to continuously monitor the setting time during AM printing, which is a disadvantage in a process that is considered heavily time-dependent.

Buildability is the most researched aspect of 3DP concrete since the ability of a printed structure to not fail under its own weight is considered an indication of a successful print. The majority of studies conducted have aimed at providing a model to predict material and stability failure and are based on the fresh state mechanical properties and/or the rheological material properties of 3D-printed concrete [14,15,16,17,18,19]. However, like flowability, there are no standardized testing methods and/or procedures that dictate these investigations. Additionally, there have been no widely accepted methods to monitor and evaluate the buildability of a structure during printing, given that this technology adds a significant amount of variability that was not found in the traditional use of concrete. This lack of means to assess and monitor real-time changes to the intrinsic properties of the material limits the usage of AM in concrete. Therefore, the addition of a real-time test method that can be used simultaneously with existing predictive models to determine the compressive strength of the concrete materials can greatly enhance the probability of a successful print. 

Based on the aforementioned gaps that are highlighted in this field, the primary goal of this study is to propose tests and methodologies to assess the printability of 3DP concrete materials. Moreover, these test methods will serve as a basis to simultaneously address the three major printability properties of flowability, setting time, and buildability.

**Figure 1:**
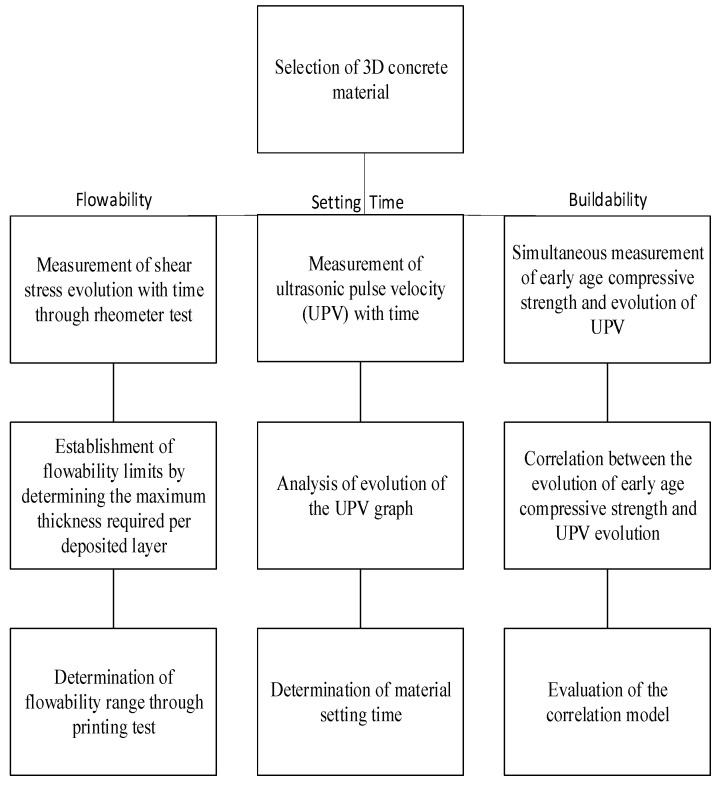
Workflow diagram of the process followed in this study.

The relationship between the evolving shear strength of a 3DP concrete material and the flowability is investigated, as the dynamic shear stress dictates the beginning of the flow of concrete until it reaches a rest position [20]. This relationship would allow for a flowability range to be set, and the limits of this range would be dictated based on user-defined maximum layer thickness and the onset of cracks/gaps in the printed layer. Consequently, this flowability range can then be used to decide on a suitable time period for printing use. Moreover, a non-destructive test that can continuously and accurately monitor the setting time is introduced by leveraging ultrasonic pulse technology and applying it on fast-setting commercially available concrete material designed for 3DP. This non-destructive test also allows for the continuous monitoring of the hardening of the material and thus can be used to monitor the effects of various additives on concrete materials. Additionally, the relationship between the ultrasonic pulse evolution of 3DP concrete material and its early-age compressive strength evolution is investigated to non-destructively predict the early-age compressive strength of the concrete material. This relationship introduces the ability to determine the compressive strength at the early stages of printing, which offers the advantage of continuously monitoring the material during printing to ensure successful fabrication. The process of achieving these goals is summarized in Figure 1 and is described in detail in the following section.

## 2. Materials and Methods

### 2.1. Materials

A commercially available printable material, CyBe MORTAR, was used to conduct the experiments as it is specially developed for 3D printing applications. CyBe MORTAR was provided by CyBe Construction B.V., Oss, The Netherlands. This material was developed to have an extremely rapid setting time of three minutes, as well as a fast compressive strength gain of 20 MPa after five hours in order to meet the demands of the additive manufacturing of concrete [21].

Retarding additives can also be used in order to extend the workability of the material by extending the setting time beyond three minutes. MasterRoc HCA 10 by Master Builders Solution Ltd., Dubai, United Arab Emirates was used in different quantities as a retarder additive for the tests. The mix compositions can be seen in Table 1, with CB-R1 representing the proportions recommended by CyBe. The mixing of the mortar took place using an Ika Mixer for quantities less than 400 g and a Hobart Mixer for quantities larger than 400 g. The mortar was first added into the mixer and dry-mixed at the low-speed setting; afterwards, the liquid materials were added over a period of ten seconds at low speed. The mixer’s speed was then raised to the medium speed setting for an additional 30 s, after which the mixer was stopped, and the mixing stage was completed. This mixing procedure differs from the ASTM C305 standard for mixing mortar, which takes up to 4 min, because of the rapid setting of the material which would see it harden if the ASTM procedure were followed.

### 2.2. Flowability Test Methods

Previous investigations on flowability focused on using empirical tests, such as the slump test, to define an acceptable range for printable material. One such study, conducted by Tay et al. [7], presented a slump value range of 4–8 mm and a slump flow range of 150–90 mm, which should define a suitable printable material with good flowability and buildability. However, these ranges do not account for the different environmental and logistical variability that can be present during a print. This would include changes to water temperature, ambient temperature, humidity, print size, and the time needed for the fresh concrete to travel from the mixing chamber to the nozzle, which depends on the size of the print. All these factors, along with the fact that the rheology of the material is ever-changing due to the hydration reaction, make it difficult to base a printability range on an empirical test. Moreover, changes to rheology are detected with more sensitivity through intrinsic properties (viscosity and shear yield strength) as compared to the slump test [11]. 

**Figure 2:**
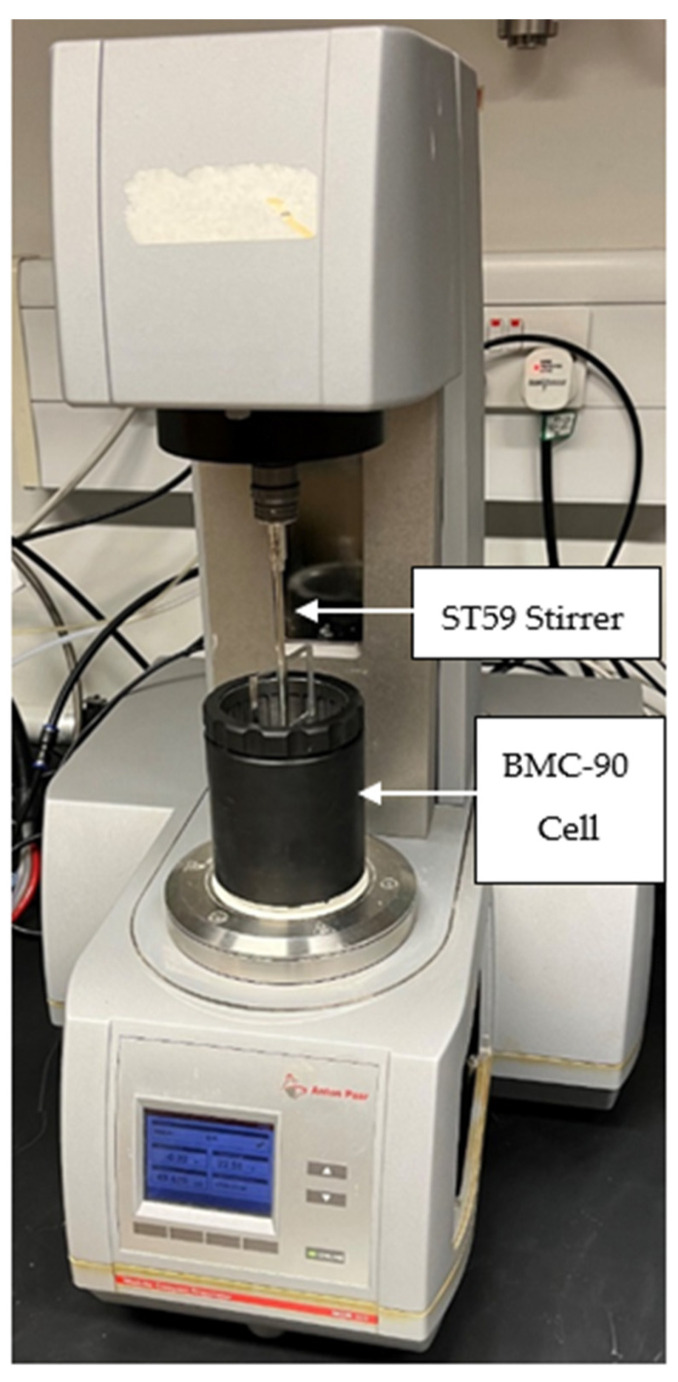
Anton Paar MCR 302 rheometer with the ST59 stirrer attachment and the BMC-90 cell.

Presuming mortar to behave according to a Bingham model material, the Anton Paar MCR302 rheometer, shown in Figure 2, was used to conduct the rheological tests, where the shear strength evolution, with respect to time, was monitored continuously. The shear strength evolution tests of mixes CB-R1, CB-R1.25, and CB-R1.5 were conducted at a speed of 1200 rpm, to replicate the mixing speed of the Putzmeister P-12 cavity concrete pump that was used to print the material. The BMC-90 cell and the ST59 stirrer attachments, shown in Figure 2, were used as they are designated for use on building materials such as cement, concrete, and plaster [22]. It must be noted that three separate trials were conducted for each mix and the arithmetic mean of the shear yield strength values, with the standard deviation, were plotted.

**Figure 3:**
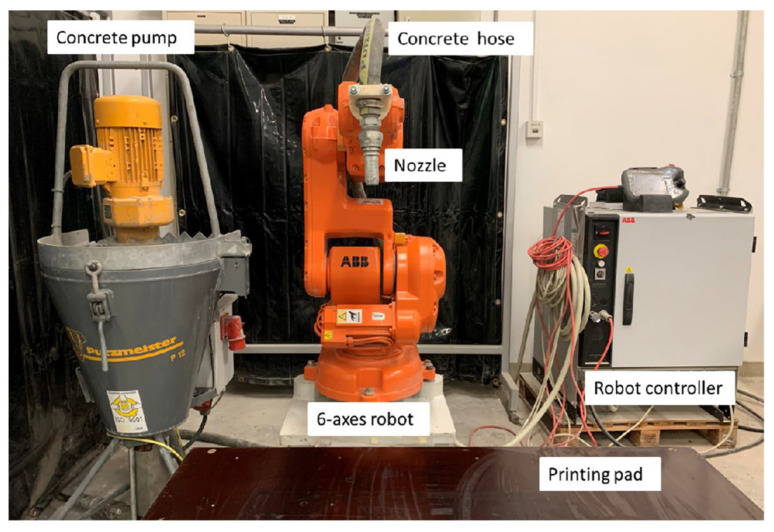
Lab-scale concrete 3D printing setup.

**Figure 4:**
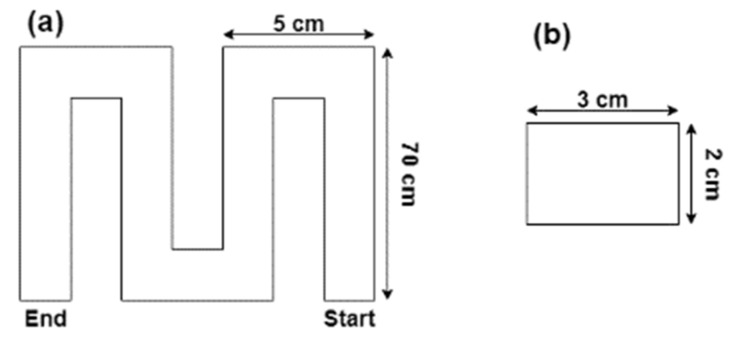
(**a**) Printing path of single layer filament print test. (**b**) Cross-sectional area of the theoretical deposited layer.

Afterwards, the mixes were subjected to printing tests to determine the shear strength-defined printability limits. A lab-scale 3D concrete printer setup was utilized, with a 6-axis ABB IRB-140 robotic arm and a Putzmeister P-12 cavity pump being used, as shown in Figure 3. The printing tests consisted of depositing single-layer filaments based on the print path shown in Figure 4a. The printer’s parameters of volumetric flow rate (v), print speed (v), and nozzle height (h) were kept constant and were 112.5 cm^3^/s, 18.5 cm/s, and 2 cm respectively. The theoretical layer thickness was calculated based on the printer parameters according to $t=\frac{\dot{v}}{v\times h}$. These print parameters can then be modified to achieve a different layer thickness, which would be determined from the required print design.

A cross-sectional view of the single-layer filament with its dimensions is shown in Figure 4b. The theoretical layer thickness was found to be 3 cm and user-defined design parameters allowed for a tolerance of 0.5 cm. Therefore, the lower limit of the printing test is defined as the instance where the layer thickness is at a maximum of 3.5 cm, which indicates the onset of an acceptable printed layer. Additionally, the upper limit is defined as the instance where cracks and gaps are visible in the printed layer, which marks the onset of an unacceptable printed layer.

### 2.3. Setting Time Test Methods

The ultrasonic wave pulse velocity (UPV) is used as a method to continuously monitor the hydration reaction of 3DP concrete and thus may be used as an alternative test to determine the setting time. UPV tests have been previously conducted on traditional mortars and concretes due to their increased sensitivity to microstructural changes and their potential for more accurately distinguishing the changes when compared to penetration tests, such as the ASTM C403 and ASTM C-191 [23,24,25,26,27,28,29,30,31,32]. Therefore, the aim of this study is to leverage the sensitivity of this test to monitor the evolution of 3DP concrete.

The ultrasonic pulse test consists of a transducer and a receiver that are in direct contact with the fresh concrete sample. The transducer emits a longitudinal pulse wave that travels across the concrete sample and is detected by the receiver on the other end of the sample. The ultrasonic pulse velocity (UPV) is then calculated using the distance between the receiver and transducer, L, and the elapsed travel time of the wave, *t_upv_*, according to $UPV=\frac{L}{t_{upv}}$ [24].

**Figure 5:**
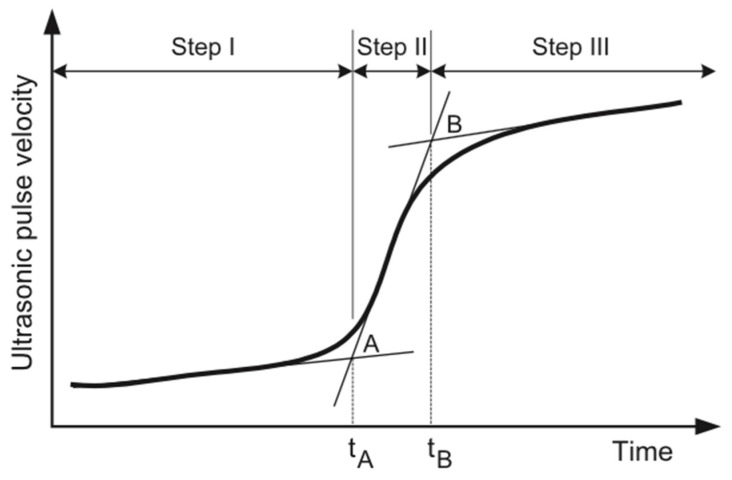
Evolution of the ultrasonic pulse velocity (UPV) of cementitious materials after mixing.

The velocity of the longitudinal wave is known to be sensitive to the formation of hydration products, which occurs during the setting of concrete [24]. As the hydration products increase in volume, the sample transforms from a viscous suspension into a porous solid, and as a result, the velocity of the longitudinal wave through the solid increases [24]. The evolution of UPV is described by the curve in Figure 5, where there is an increase in velocity with time. The curve is divided into three distinct phases, where phase I represents the viscous suspension state that the sample is initially in. During this phase, the hydration products begin to form and eventually lead to the percolation of cement grains, which represents the start of the transformation of the sample from a viscous suspension to a porous solid [23]. Towards the end of phase I, the rate of hydration product formation increases, and a sharp increase in the UPV is observed, which signals the beginning of phase II. During phase II, the percolation of more cement grains continues, and the porous solid structure becomes further interconnected as the pores that were present in the viscous suspension start to fill with hydration products, which results in an increase in the compressive strength of the material. As the average pore size decreases, a sharp increase in the rate of UPV gain can be noticed in Figure 5. Afterwards, the UPV values level off and approach an asymptotic value for the final concrete structure, which signals the end of phase II. Phase III represents the stage where the structure’s transformation into a porous solid has reached a slow, steady state. This phase represents the final stage of the 3D-printed concrete, where most of the hydration is completed and the pores have largely filled with hydration products [25].

After this stage has been reached, the concrete structure continues its compressive strength gain over the following days and weeks. Previous studies conducted on cementitious materials have identified characteristic points on the UPV that correlate with the setting times of these materials [25,26,27,28,29,30]. These characteristic points are points A and B in Figure 5, which represent the inflection points of the graph, as well as the transition from one step to another. The first inflection point A was found to represent the initial setting time for various cementitious pastes, mortars, and concrete [25,30,31]. The final setting time was found to correlate to a point between the maximum rate of UPV increase and point B. However, this relation is not as directly clear as the relation between the initial setting time and point A [14,25,26]. For 3DP concrete or mortar, the setting process is often accelerated significantly versus normal concrete, with the timeline between initial and final sets significantly compressed. Thus, the focus of this paper is to investigate the feasibility and reliability of the UPV–setting time relation on 3D-printed concrete mixtures for their use as an in situ monitoring procedure for the quality control of the printed material.

**Figure 6:**
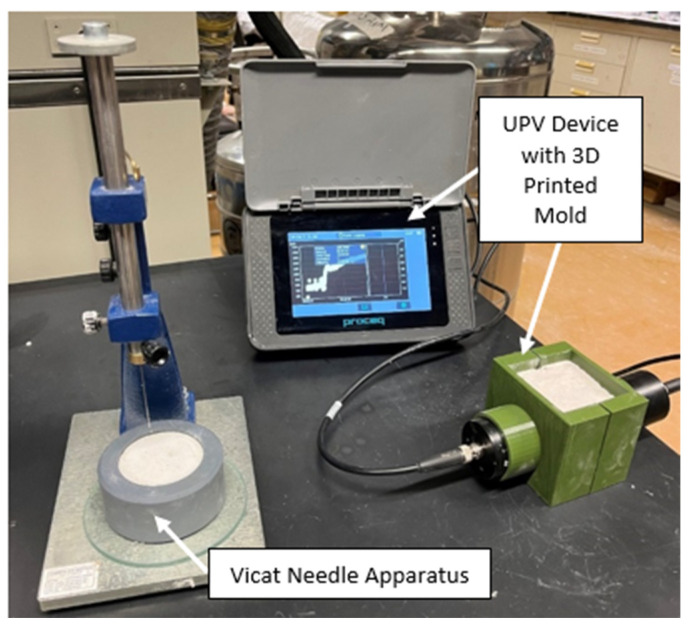
Experimental setup used to measure the setting time.

The Pundit Pl-200 with the 54 kHz transducer/receiver by Proceq SA, Schwerzenbach, Switzerland was used to measure the evolution of the UPV of the specimens, as shown in Figure 6. The 54 kHz transducer/receiver has a minimum lateral dimension constraint of 69 mm, thus a 70 mm × 70 mm square 3D-printed ABS mold was used as a housing element for the transducer and receiver. Once the fresh concrete is mixed, it is poured inside the mold, where it is in direct contact with the transducer and receiver. The Pundit Pl-200 then records the UPV value of the concrete specimen every 5 s, and the evolution of the UPV curve is determined. This UPV evolution curve is then studied and the first inflection point that corresponds to the setting time is determined. The setting time that is determined from the UPV curve is then compared with the setting time that is determined from the ASTM C-191 for the same mix. The ASTM C-191 initial setting time was obtained by using a Humboldt Mfg. Vicat needle apparatus, as shown in Figure 6. However, the testing procedure was altered to find the penetration of the needle continuously at every 30 s mark after the specimen rests in the mold undisturbed for 3 min. This frequency of penetration was increased from the standard (penetration frequency every 15 min after the specimen rests in the mold undisturbed for 30 min) due to the fast-setting nature of the tested specimens. The setting time was then found using:

$$Setting\;Time=\left(\left(\frac{H-E}{C-D}\right)\times \left(C-25\right)\right)+E$$

where *H* is the time in minutes of the last penetration greater than 25 mm, E is the time in minutes of the last penetration less than 25 mm, C is the penetration reading at time *E*, and *D* is the penetration reading at time *H* [33]. Three separate trials of the UPV test and the Vicat needle test were conducted for each mix and the arithmetic mean was recorded along with the standard deviation.

### 2.4. Buildability Test Methods

Buildability is generally defined as the ability of a 3D-printed layer of concrete to withstand its own weight and the weight of subsequently deposited layers [15,16,19,34]. Printing failure due to insufficient buildability is attributed to either plastic collapse, stability failure, or both. Moreover, the thickness deformation of a printed layer after the addition of subsequent layers, i.e., shape stability, is another important aspect of buildability. Shape stability may be defined as the maximum allowable user-defined deformation of a deposited layer upon the addition of external forces from subsequent layers. Plastic collapse is defined as material failure due to the compressive load,$\;\sigma_{C}$, exceeding the material’s own compressive strength, $\sigma_{M}$, [15] such that $\;\sigma_{C}=\rho gh\left(t\right)$, where ρ is the density of the material, g is the gravitational acceleration constant, and *h*(*t*) is the height of the printed component as it varies with time. Stability failure, on the other hand, is a consequence of the local or global instability of the printed structure that results in buckling and thus the failure and collapse of the structure [17,18]. This mode of failure is theoretically in effect when the elastic Young’s modulus of the material is less than a critical value, $E_{C}$, [15] which is defined by $E_{C}\approx \frac{\rho gA{\left(h\left(t\right)\right)}^{3}}{8I}$, where *A* is the horizontal cross-sectional area, and *I* is the quadratic moment of inertia. It is important to note that the derivation of this relation assumes that Young’s modulus is uniform throughout the structure, which would not exist in a 3D-printed structure. However, this approximation has shown that it underestimates the ability of a structure to resist buckling and therefore can be used as a conservative model [35].

For both modes of failures, the governing material properties (i.e., compressive strength and elastic Young’s modulus) of concrete are time-dependent and thus evolve during the printing process. Various studies have aimed at providing a model to predict material and stability failure, with these models being based on the mechanical properties and/or the fresh-state rheological material properties of 3D-printed concrete [18,19,34,35,36]. In this study, the evolution of the compressive strength of the 3D-printed concrete is monitored and its relationship with the UPV graphed in Figure 5 is investigated. The objective is to monitor the early-age compressive strength of the material to be able to use the existing predictive models to determine the printing limitations of a certain material. Moreover, the relation between the evolution of compressive strength with the evolution of the UPV is investigated to determine whether UPV can be used as a tool to non-destructively predict the compressive strength of the concrete material. It should be noted that there have been studies that have investigated the relation between UPV and the compressive strength of concrete before, however, these investigations have focused on predicting the compressive strength between 1 day and 180 days [37,38,39]. Moreover, the evolution of the UPV graph was not taken into consideration, as the relations found in these studies focused on the long-term compressive strength gain of concrete materials. In this study, the relation between the early-age (between initial setting and less than 1 hr since mixing) compressive strength of the 3D-printed concrete and UPV is investigated, since that is the period where plastic collapse would occur during printing. Additionally, the rate of increase in the UPV values for fast-setting concrete is much higher than the traditional concrete used in previous studies. It takes the CyBe mortar used in this study only 30 min to reach a UPV value of more than 3000 m/s; however, it would take traditional concrete 24 h to reach that value [37]. Therefore, the discrepancies in the rate of increase in the UPV also result in discrepancies in the predicted compressive strength.

The MATEST E160N compression testing machine was used to determine the early-age compressive strength of the samples through the ASTM C109/C109M standard at a loading rate of 900 N/s [40]. The only deviation from the ASTM standard was done for the time of testing, where the compressive strength was recorded at 10, 11, 13.5, 15, 20, 26, 30, 35, and 45 min after mixing in order to monitor the early-age compressive strength gain as accurately as possible. A simultaneous UPV test was conducted on the same batch of concrete mix to correlate the compressive strength gain and the evolution of the UPV of the specimen. Three separate trials were conducted for each testing time. The arithmetic mean and standard deviation were determined at each testing time.

## 3. Results and Discussion

### 3.1. Flowability

**Figure 7:**
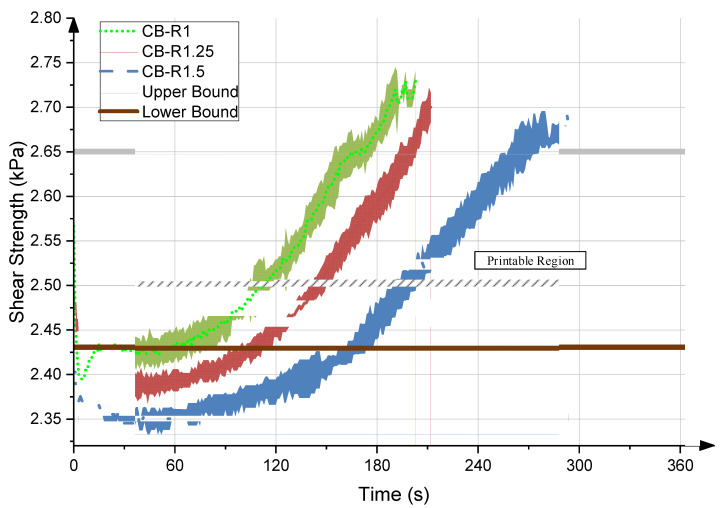
Evolution of shear stress for mixes 1, 2, and 3.

Mixes CB-R1, CB-R1.25, and CB-R1.5 were used to determine the shear yield strength evolution of the material with time. As discussed earlier, different retarder quantities were used to determine the sensitivity of the test and the effect on the shear yield strength evolution. The resulting shear yield strength evolution graphs are shown in Figure 7. As would be expected, the addition of a higher dosage of retarder delays the evolution of the graph and thus extends the time allowed for the material to be printed. It was also noticed that as the retarded quantity increased, the initial shear yield strength values decreased, which is due to the delayed onset of the hydration reaction.

**Figure 8:**
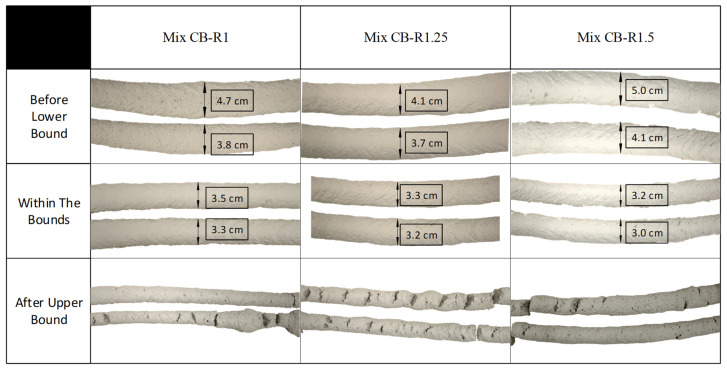
Printed single-layer filaments for mixes 1, 2, and 3 at various stages of printing.

Afterwards, printing tests were conducted on each of the mixes to determine the printability range based on the pre-defined lower and upper bound conditions. The initial conditions of the rheological tests were replicated, where a minute and a half of mixing was performed before the pumping of the material was started. Single-layer filaments were deposited, and the thickness of the layers was measured, with 3.5 cm representing the onset of an acceptable layer. The time corresponding to the first acceptable printed layer was used to define the corresponding lower bound of the printable shear strength range. This lower bound can be adjusted for different prints based on design requirements and printing parameters. The mixes were continuously printed up until cracks and gaps started to be present in the layers, and the time corresponding to the onset of the flaws was used to define the corresponding upper bound of the printable shear strength range. The single-layer filaments at various stages of printing are shown in Figure 8 along with their corresponding layer thickness. The determined upper and lower bounds were plotted on the shear strength evolution graphs, as shown in Figure 7, and a printable region was found for the material.

### 3.2. Setting Time

The evolution of the UPV with time was monitored for mixes CB-R1, CB-R1.25, CB-R1.75, and CB-R2. The quantity of the retarder additive used varied in mixes CB-R1, CB-R1.25, CB-R1.75, and CB-R2 to monitor the sensitivity of the tests with changing setting times. The UPV measurements were initiated 1 min and 30 s after the start of mixing to allow time for mixing as well as the filling of the mold that houses the transducer/receiver. Simultaneously, the setting time of these mixes was measured using the Vicat needle apparatus in order to compare them with the setting time values obtained through the UPV test.

**Figure 9:**
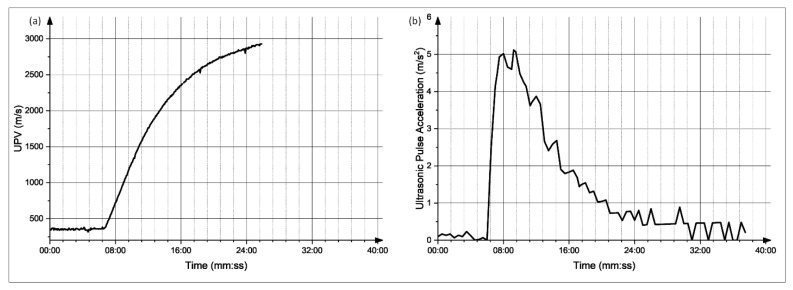
(**a**) Evolution of UPV with time for mix 1. (**b**) Derivative of the UPV graph.

**Figure 10:**
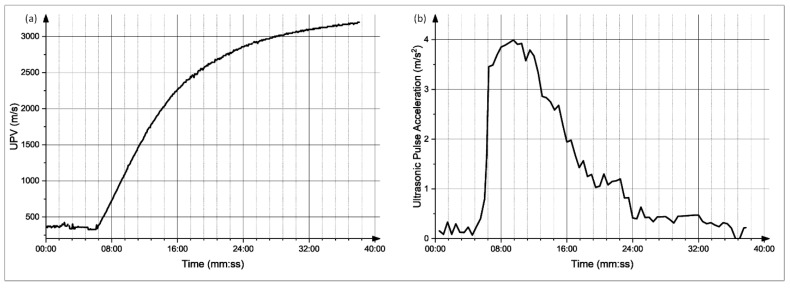
(**a**) Evolution of UPV with time for mix 2. (**b**) Derivative of the UPV graph.

**Figure 11:**
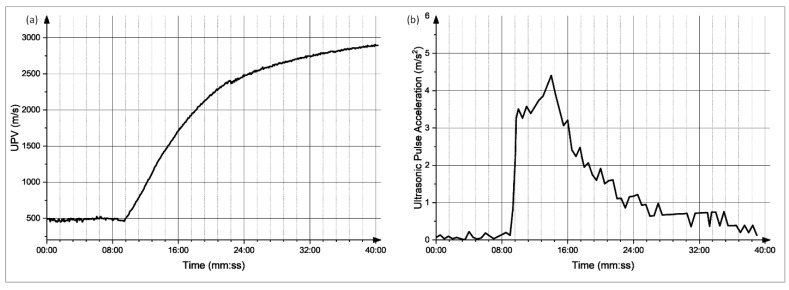
(**a**) Evolution of UPV with time for mix 4. (**b**) Derivative of the UPV graph.

**Figure 12:**
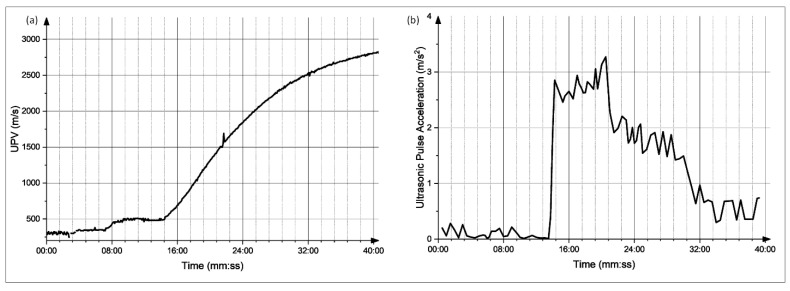
(**a**) Evolution of UPV with time for mix 5. (**b**) Derivative of the UPV graph.

The graphs obtained for each of the mixes were analyzed; moreover, the derivative of these graphs was also obtained. The setting time was determined at the point of a sudden increase in the UPV values, which is the time at which there is a major spike in the UPV derivative plot. The UPV graphs of the CyBe Mortar mixes, as well as their corresponding derivative plots, are shown in Figure 9, Figure 10, Figure 11 and Figure 12. Moreover, the graphs and the setting time values obtained showed sensitivity to an increased retarder dosage as the setting time values increased with an increased retarder dosage. Therefore, the sensitivity of this test to factors affecting the hardening of the material is shown. The setting times found through the UPV test and through the ASTM C-191 Vicat needle test can be found in Table 2.

The initial setting time values extracted from the UPV test were found to be similar to the values obtained from the Vicat needle test, with the largest difference between the reported values being 4.9%. This high similarity is indicative of the practicality of using the UPV test as an alternative, non-destructive test that can be used to continuously monitor the setting of concrete. Moreover, the UPV test could be a better alternative for setting time measurements as the ultrasonic wave is transmitted throughout the entirety of the material and, as a result, can reflect internal microstructure changes more adequately than a penetration test, which is more sensitive to surface properties. The high similarity in the reported values exceeds the best similarity the authors found in the literature for cementitious materials, which was 17% [9]. This may suggest that the fast-setting nature of 3D-printed concrete is better suited for using the UPV test to identify the initial set than traditional cementitious materials. This can be attributed to 3D-printed concrete’s evolution of the microstructure near the surface of the concrete and in the core of a sample, in which the microstructure evolutions tend to be similar; whereas, for traditional concrete, the temperature gradients and drying conditions result in different microstructure evolution rates in the core and near the surface. Thus, the alignment of the setting time identified by the UPV test, which is influenced by the entire sample microstructure, including the core, and Vicat test, which is influenced by the sample surface only, should be more consistent for faster-setting concrete.

### 3.3. Buildability

**Figure 13:**
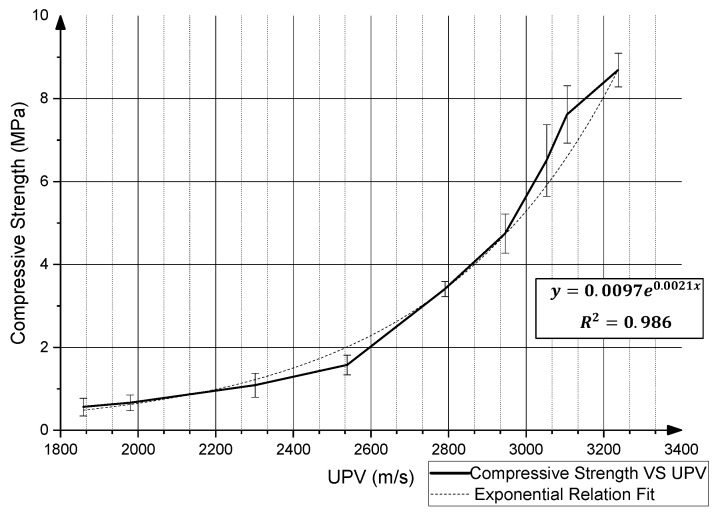
Plot of early-age compressive strength VS UPV for mix 2.

Mix CB-R1.25 was used to determine the early-age compressive strength’s relationship with the UPV evolution, since its initial setting time would allow for a 6 min period of mixing and mold filling. Nine 50 mm × 50 mm square specimens were prepared to be tested at the specified times. Alongside the nine cubical specimens, the 3D-printed mold was also filled with the same batch of material to monitor the UPV evolution. The experimental results of the compressive strength tests and their corresponding UPV values (m/s) are reported in Table 3, where the data are a result of the average of four trials that were conducted. These results were plotted against each other in Figure 13, and an exponential relationship was found between both quantities through the following equation: $\sigma_{C}=0.0097\left(MPa\right)e^{\left(0.0021\left(\frac{s}{m}\right)\right)\left(UPV\;\left(\frac{m}{s}\right)\right)}$, where $\sigma_{C}$ (MPa) is the predicted compressive strength value.

Afterwards, the exponential relation was tested by predicting the compressive strength of another set of mixed CB-R1.25 specimens and comparing the predicted values with the actual compressive strength of these specimens. These values, along with the corresponding UPV values, are reported in Table 4, and it was found that exponential relation yields a maximum percentage difference of approximately 13% and a maximum absolute difference of 0.65 MPa. Therefore, it can be noted that a material-specific relation can be found between the early-age compressive strength and the UPV reading that would assist in non-destructively monitoring and predicting the early-age compressive strength of 3D-printed concrete. The presence of such a relation could be significant towards implementing a successful print, since the early stages of printing are the most pivotal, with the material not yet gaining most of its final strength. Therefore, the early-age compressive strength dictates the feasibility of certain designs being completed successfully. It must be noted that due to the limited sensitivity of the evolution of the UPV with respect to the long-term gain of compressive strength, the aforementioned relation would only target the early-age compressive strength of the material. Here, early age denotes the time period in which the UPV values start increasing, which is the period right after the setting of the material up until the UPV values reach a constant value, where the graph plateaus and no correlation can be made with the gain of compressive strength.

## 4. Conclusions

This study introduced test methods that form the basis for the development of a framework for the characterization of the printability of cementitious materials based on three controlling properties: flowability, setting time, and buildability. These properties are considered the minimum requirements needed for a printable material and thus identifying, monitoring, and characterizing them is a vital step in introducing this framework. 

Flowability is assessed by monitoring the evolution of shear stress as a function of time using a rheometer. Consequently, a shear yield strength-based range was determined such that the material is printable based on the flowability requirements. These requirements are defined based on the ability of the material to be pumped and extruded without any breakages and disruptions in the flow while retaining its deposited shape. 

The study has also shown that the UPV test can be used to determine the initial setting time of 3D-printed concrete. It was shown that the setting time of various mixes as determined by the proposed UPV test correlates well with the results of the ASTM C-191 standard (e.g., penetration by the Vicat needle). Moreover, the UPV test is a better alternative to the ASTM C-191 standard as it considers setting over a spatially averaged thickness, while the standard penetration test evaluates a highly localized region. Furthermore, the UPV was also found to be useful in predicting the early-age compressive strength of 3D-printed concrete, where a material-specific relation can be found with a maximum of 13% difference found between predicted and measured values. This UPV-compressive strength relation could be used along with existing models in order to predict the buildability failure.

Future work will focus on leveraging the initial setting time of the material to determine appropriate deposition times and ideal structural integrity. The work will also focus on expanding the buildability property by appending tests focused on characterizing the shape stability of the material. The implementation of the test methods on different classes of 3D concrete is essential in refining and improving the outcome of these methods. Additionally, comprehensive printability maps can be established by integrating the effects of process parameters (e.g., printing speed, pressure, nozzle diameter) with the test methods and properties presented in this study. 

## Figures and Tables

**Table 1:** Mortar mixture proportions.

Mix #	Mix Name	CyBe MORTAR (g)	Water (g)	Retarder (g)
1	CB-R1	800	128	1.00
2	CB-R1.25	800	128	1.25
3	CB-R1.50	800	128	1.5
4	CB-R1.75	800	128	1.75

**Table 2:** Setting times for different mixes using the UPV test and Vicat needle test.

Mixes	UPV Time (Min)	Vicat Needle Time (Min)	% Difference
CB-R1	8.2 ± 0.8	8.5 ± 0.6	3.5
CB-R1.25	8.8 ± 0.6	8.6 ± 0.9	2.3
CB-R1.75	11.3 ± 1.1	10.7 ± 1.0	4.7
CB-R2	15.7 ± 0.6	16.2 ± 0.7	3.0

**Table 3:** Experimental compressive strength values of mix 2 with their corresponding UPV values.

Time (Min)	Compressive Strength (MPa)	UPV (m/s)
10	0.56	1858.8
11	0.66	1979.5
13.5	1.08	2301.8
15	1.57	2539.0
20	3.40	2791.2
25	4.74	2945.9
30	6.50	3052.7
35	7.61	3105.8
45	8.69	3237.4

**Table 4:** The predicted and actual compressive strength values with their corresponding UPV values.

Time (Min)	UPV Values (m/s)	Predicted Compressive Strength Values (MPa)	Actual Compressive Strength Values (MPa)	Percentage Difference (%)
10	1786.3	0.41	0.44	5.7
11	1876.4	0.50	0.49	1.6
13.5	2127.2	0.85	0.92	8.0
15	2356.9	1.37	1.42	3.8
20	2723.7	2.96	2.73	8.2
25	2916.7	4.44	4.43	0.1
30	3030.3	5.63	4.98	12.9
35	3139	7.07	7.48	5.5
45	3223.4	8.44	8.77	3.7

## Data Availability

Not applicable.
